# Coracobrachialis Longus Muscle: Humeroepitrochlearis

**DOI:** 10.7759/cureus.2615

**Published:** 2018-05-13

**Authors:** Georgi P Georgiev, R. Shane Tubbs, Boycho Landzhov

**Affiliations:** 1 Department of Orthopaedics and Traumatology, Medical University of Sofia, Bulgaria, University Hospital Queen Giovanna, Sofia, BGR; 2 Neurosurgery, Seattle Science Foundation, Seattle, USA; 3 Department of Anatomy, Histology and Embryology, Medical University of Sofia, Bulgaria, University Hospital Queen Giovanna, Sofia, BGR

**Keywords:** coracobrachial variations, humeroepitrochlearis muscle

## Abstract

During a routine anatomical dissection of the right brachium of a 68-year-old male cadaver, an extremely rare variation of the coracobrachialis longus muscle was discovered. It started from the medial surface of the middle part of the humerus with a well-formed muscle portion and then continued into the well-presented distal tendinous portion, which was attached to the medial epicondyle of the humerus. We also briefly review the reported variations of the coracobrachialis and their potential clinical significance.

## Introduction

The coracobrachialis muscle (CB) classically originates from the apex of the coracoid process, together with the short head of the biceps brachii, and inserts on the medial surface of the shaft of the humerus between the attachments of the triceps and brachialis [1]. It is perforated and innervated by the musculocutaneous nerve [2]. The CB flexes the arm medially forward and abducts together with the anterior fibers of the deltoid, maintaining the head of the humerus within the glenoid fossa [2].

In most species, the CB has three portions: the CB longus, CB medius and CB brevis. In humans, the medius and longus fuse to form the CB [2].

In 1867, Wood first observed that the CB had three parts: the upper part starts from the coracoid process and is attached to the capsule of the shoulder joint (CB superior or brevis or rotator humeri); the middle part is inserted into the mid-part of the humerus and is observed in humans (CB proprius or medius); the lower part is attached to the internal condyloid ridge, the internal intermuscular septum, or the trochlea (CB longus) [3]. Kyou-Jouffroy et al. (as cited by Catli et al.) also described three parts of the CB starting from the coracoid process and inserting into the medial epicondyle of the humerus (CB longus or superficialis), humeral diaphysis (CB medius), and humeral neck (CB profundus or brevis) [4]. In contrast, Mori e al. [5] described the CB as divided into superficial and deep layers in 16% of Japanese and incompletely divided in 8%.

Anatomical variations of the CB have seldom been described in the anatomical and surgical literature [3,6-8]. However, some of them could have definite clinical significance [9].

In this case report, we present an extremely rare variation of the CB longus muscle, termed the humeroepitrochlearis, and briefly review the reported variations of the CB and their potential clinical significance.

## Case presentation

**Figure 1:**
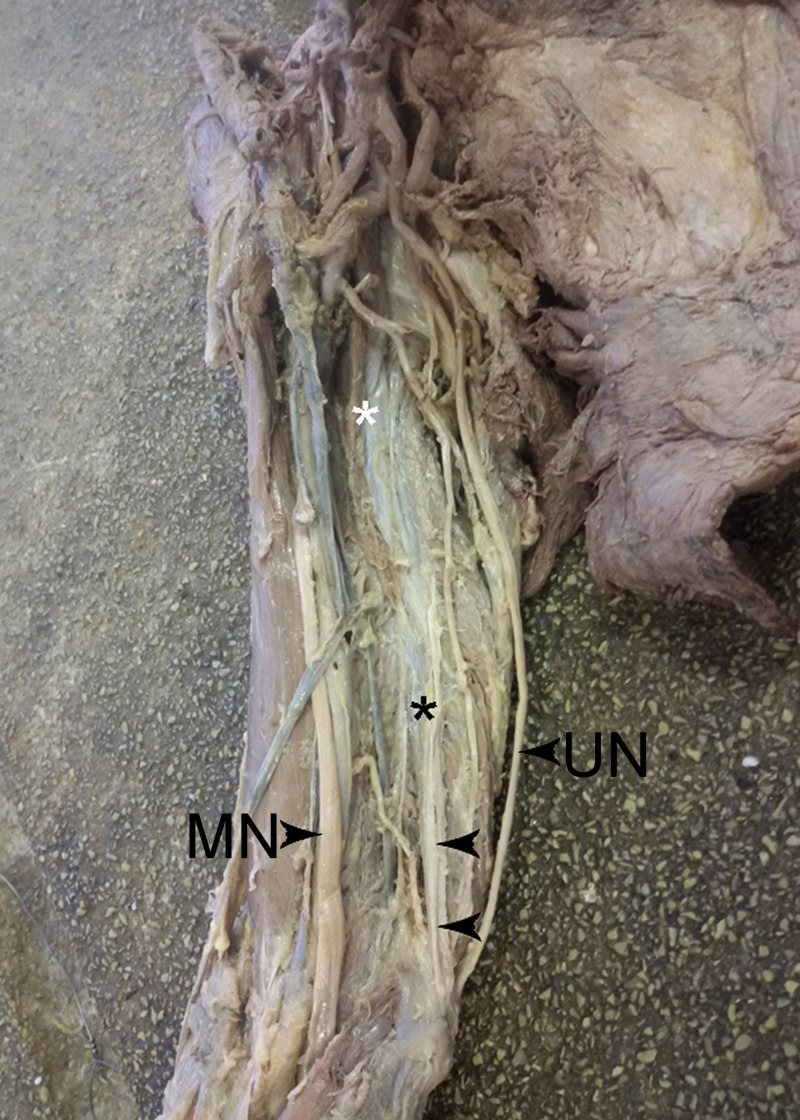
The right brachium Muscles: Humeroepitrochlearis muscle body (black asterisk); classical coracobrachialis muscle (CB) (white asterisk); Humeroepitrochlearis muscle body (black arrows). Nerves: UN, ulnar nerve; MN, median nerve.

A rare case of a CB variation was observed during a routine anatomical dissection in the right arm of a Caucasian male cadaver aged 68 years at death. The dissection was approved by the Medical-Legal Office and Local Ethics Committee. The proximal part of the CB corresponded to the classical description of this muscle, taking origin from the apex of the coracoid process together with the short head of the biceps brachii. The muscle then inserted in the medial surface of the shaft of the humerus between the attachments of the triceps and brachialis. This part continued as a thin fibrous layer with the second part of the muscle, which started from the medial surface of the middle part of the humerus with a well-formed muscle portion (length – 46 mm, maximal width – 14 mm), and then continued into the well-presented distal tendinous portion (length – 68 mm, maximal width – 3 mm), which was attached to the medial epicondyle of the humerus (Figure 1).

The variant muscle was innervated by the musculocutaneous nerve. No medical or surgical history of the cadaver was available. There was no similar variation in the contralateral upper limb.

## Discussion

Different variations of the CB, especially of CB longus, were mainly reported in the old anatomical literature (Table 1).

**Table 1:** Reported variations of the CB muscle CB: coracobrachialis

Variations and terms	Reference
Three-headed CB (CB brevis, normal CB, and coracoepitrochlearis muscle)	Georgiev et al. [9]
Proximal accessory bands to the lesser tubercle or surgical neck of the humerus (CB superior, CB brevis, or CB profundus)	Wood et al. [3], Kyou-Jouffroy et al. [4], Bergman et al. [6], Bauones et al.[10]
Proximal accessory bands started from the conoid ligament of the clavicle and inserted into the medial intermuscular septum	Chouke et al. [2]
Proximal accessory bands to the capsule of the shoulder joint (coracocapsularis)	Wood et al. [3], Bergman et al. [6]
Proximal accessory bands to the pectoralis major tendon (coracobrachialis minor secundus)	Wood et al. [3], Bergman et al. [6]
Proximal accessory bands to the tendon of the latissimus dorsi (CB brevis s. rotator humeri, le court coracobrachialis, minor coracobrachial muscle of Cruveilhier)	Bergman et al. [6]
The medial head of the triceps	El-Naggar et al. [7]
The brachial fascia	Ilayperuma et al. [1]
Distal insertion to the medial supracondylar ridge (CB inferior or CB longus)	Wood et al. [3], Bergman et al. [6]
Distal insertion to medial intermuscular septum trochlea or supracondylar process (CB inferior or CB longus)	Wood et al. [3], Bergman et al. [6]
Distal insertion to the medial epicondyle of the humerus or the antebrachial fascia (CB inferior, CB longus and CB superficialis)	El-Naggar et al. [7]

The numerous terms used to describe them have confused anatomists and surgeons alike. In order to simplify the reported variations and terms, Georgiev et al. [9] suggested dividing them into two simple groups: (a) CB brevis, inserted into the proximal part of the humerus, and (b) CB longus, inserted into the distal brachium. They do not use the term CB medius and accept this as the classically described main CB muscle. Georgiev et al. [9] also propose the term “coracoepitrochlearis muscle” to denote a variant CB muscle that originates from the coracoid process and inserts into the medial epicondyle of the humerus. According to this classification, the accessory superior part in our case can be regarded as classical CB. The lower part, termed the “humeroepitrochlearis” muscle, belongs to the second group.

The CB variants are described as deriving embryologically from the lateral mesoderm along with the other upper limb muscles. The muscle primordia fuse to form a single muscular body, which then regresses as the layers of arm muscles differentiate. The presence of an accessory CB could be explained as a result of the premature termination of this regression process [7].

Although the CB muscle is said to be functionally unimportant, it has definite clinical significance. During a blockade of the brachial plexus, the CB serves as a guide for the axillary artery [1]. It is well vascularized and a good choice as a transplant for treating long-standing facial palsy [7], as a graft for post-mastectomy reconstruction, and in both axillary and infraclavicular deformities [7]. In some cases, an accessory CB can lead to subcoracoid impingement and nerve palsy of the musculocutaneous, median, or ulnar nerves due to hypertrophy, traumatic injury, or brachial artery compression [6,8,11-12]. According to Georgiev et al. [9], the absence of clinical reports for neuro-vascular compression due to CB longus can be explained first by the rarity of the variation and, second, by the limited skin incision performed during decompression surgery; therefore, the variation cannot be revealed so it is not clearly defined. Moreover, variant CB could also cause confusion during surgery and imaging evaluation [9-10,12].

## Conclusions

The reported coracohumeral muscle could prove clinically important during a surgical approach after a traumatic injury or decompression in this region. It could also impede different imaging modalities. Moreover, it could serve as an ideal tendinous graft donor because there should be no functional loss of the extremity after it is excised.
